# Why we should contain the "medical-industrial-media complex"

**DOI:** 10.1590/S1516-31802004000200001

**Published:** 2004-03-01

**Authors:** Paulo Andrade Lotufo

In the 1950s, the general and then president of the United States, Dwight D. Eisenhower, coined the term "military-industrial complex" to indicate his preoccupation regarding the power jointly wielded by the military and the arms industry in manipulating that country’s budget planning to favor their projects for constructing new arms. Following this example, in the 1980s the editor of The New England Journal of Medicine coined the term "medical-industrial complex" to indicate the growing and equally worrying association between doctors and industrial sectors, especially the pharmaceutical industry. This fear of the relationship between medical science and companies may only have been an Anglo-Saxon and Protestant variation of the traditional Latin and Catholic rejection of profit, but it has proven to be prophetic of the reality of medical attendance, especially in Brazil. If this is doubted, let us look at just four examples.

1. A pharmaceutical company launched a campaign on billboards and shopping center display stands to warn of the danger of deep vein thrombosis. It promoted a series of talks showing the importance of its extremely expensive product and was able to substitute this product for non-fractioned heparin, in the prophylaxis of thromboembolism in all hospitals. There is no evidence that its product is superior to heparin. Even the death of the journalist Roberto Marinho, at the age of 98 years, was exploited by the company’s press liaison office to show the importance of its medication for avoiding pulmonary thromboembolism.
2. The same company launched a campaign on billboards and posters throughout the city in relation to the control of diabetes. It proposed the utilization of glycated hemoglobin, the most expensive test for diabetes control, in order to promote its new formulation of insulin, which, to the surprise of any well-informed doctor, will serve for the control of all diabetics. A weekly magazine put the carton of the product on its front cover, announcing the arrival of a new world for diabetics!
3. In gynecology congresses, there are various symposia organized by the pharmaceutical industry to publicize and stimulate hormone replacement therapy, despite the results from the most important clinical trial relating to primary prevention, the Women’s Health Initiative, which have shown that hormone replacement therapy increases the risk of cardiovascular disease, breast cancer and Alzheimer’s disease, and does not provide an improvement in quality of life.
4. Another weekly magazine published an edition with frontcover material about checkups, showing various types of equipment that are still novel in the country, which will be able to detect any cardiovascular disease or type of cancer. This was in the same week that equipment companies were removing tomography scanners from private radiological clinics or philanthropic hospitals that were unable to pay for them.

On the other hand, in the real world, far from the pyrotechnics of the media, this is the situation we face: private hospitals in difficulties because of the restrictions on payments imposed by private health funds and health insurance companies. Philanthropic and state hospitals are also in financial difficulties as a result of insufficient disbursement from the National Health System and the state and municipal budgets to cover the funding of such institutions. Along the same line of difficulties, health insurers and health funds are no longer able to pass on the rises in hospital costs to insured individuals and health fund contributors. In the same way, the public sector is now levying a tax burden that is the maximum attainable and the revenue distribution to the healthcare sector has now reached its limit. Or in other words, the resources are finite, whether in the private or public sector, whereas the voracity of pharmaceutical and equipment companies is infinite.

If the medical-industrial complex previously made use of opinion-formers to publicize its products, today it induces consumption through reports and interviews in newspapers and on radio and cable television programs every day, emphasizing the need to prevent everything and treat everything. The editorial office of *Diagnóstico & Tratamento* receives dozens of "suggestions for the agenda" from countless press liaison companies, most of which with inadequate and erroneous information on equipment, medications and medical advice. Some colleagues send us press releases that they ask us to kindly publish in our journal.

The journalist and media watcher Alberto Dines, a former editor of *Jornal do Brasil* and *Folha de S. Paulo*, has discussed this matter and named it the "Court of Miracles", because "every day, the most absurd novelties manufactured in pharmaceutical industry marketing offices, in university research departments (always short of funds) and even in generalist scientific publications (desperate to get regular advertisers) are poured out over readers, TV viewers and listeners. Since this factory of illusions is set up abroad, more precisely in New York, the mother and stepmother of all fashions, such production automatically gains the seal of authenticity and truth [here in Brazil]".^1^

So what can we do to contain the advance of the medical-industrial-media complex?

1. Implement within the medical profession the control devices utilized in other countries that have already been initiated through the Resolutions 1595/00, dated May 18, 2000, from the Conselho Federal de Medicina (National Medical Board),^2^ and RDC 102, dated November 30, 2000, from the Agência Nacional de Vigilância Sanitária (National Agency for Food and Drug Control),^3^ for the purposes of publications and the presentation of talks and speeches.
2. Establish that the responsibility for continuing medical education lies with universities and university and teaching hospitals, with the creation of forms of credit for the purposes of professional certification, admission and advancement.
3. Delegate the prerogative to bestow the title of specialist to the respective specialist societies, for the 52 specialties approved by the Associação Médica Brasileira (Brazilian Medical Association), the Conselho Federal de Medicina (National Medical Board) and the Comissão Nacional de Residência Médica (National Medical Residency Board), thereby avoiding the appearance of specialties of convenience.
4. Make it obligatory for all hospitals to have pharmacology and therapeutics committees with specific training in the analysis of clinical tests, and strengthen the committees that already exist.
5. Initiate understandings with the Conselho Nacional de Auto-Regulamentação Publicitária (National Council for Self-Regulation in Advertising) and consumer protection entities for the control of abusive and unethical advertising in this field.
6. Contact journalistic and advertising bodies to demonstrate the harmful extent of disguised and abusive advertising in the healthcare field.
7. Establish a direct channel for dialog with the pharmaceutical companies that do research, in order to encourage good clinical practices in all activities, with the strengthening of joint discussion forums such as the First Meeting of Research Ethics Committees of the State of São Paulo.

Finally, doctors need to incorporate the notion that resources destined for healthcare, whether this is state or private care, are finite. Abuse in the prescription of expensive medications and the use of costly equipment will represent a diminution of salaries and fees.
